# Meeting in Mind and a Smile on the Face: A Tribute to Dr. Randall J Cohrs

**DOI:** 10.3390/v14061124

**Published:** 2022-05-24

**Authors:** Jayasri Das Sarma

**Affiliations:** 1 Department of Biological Science, Indian Institute of Science Education and Research—Kolkata, Mohanpur 741246, India; dassarmaj@iiserkol.ac.in; 2 Department of Ophthalmology, University of Pennsylvania, Philadelphia, PA 19104, USA

It is my privilege to have a mentor cum friend like Prof. Randall Cohrs, who played a significant role in mentoring me and moulding me into the scientist and mentor that I am today. I first met Randy at the 9th International Symposium on Neurovirology held in 2009 in Miami, FL, USA. Cohrs’s then mentor and dear friend Dr. Donald H Gilden introduced me to him. Randy being a kind and generous human, we became friends in no time. Although by training and practice, he was a Herpes virologist and I was a Corona virologist, we often found ourselves discussing science over coffee. Our friendship made me realize that friendship knows no boundaries of age, ethnicity, language, and the branch of science you chose to pursue.

I had the chance to meet Randy again in 2010 when I was invited to deliver a grand round lecture at the Department of Neurology, University of Colorado by Dr. Gilden. Randy was a very compassionate human being; and this meeting further strengthened our friendship.

Randy was a mentor to me; he taught me an essential skill for a scientist in this era: networking and conducting meetings. In 2013, Randy encouraged me to apply for the Indo-US science and technology forum (IUSSTF) American Society for Microbiology (ASM) professorship award. We wrote the grant together, which was funded, and Randy served as my mentor for the program. This was our first venture into collaboration together and a very successful one, highlighted on the International Society of Neurovirology (ISNV) and Indo-US website (Figure 1). This collaboration gave me a sense of trust and confidence in my mentor and myself. I found myself discussing my challenges as a scientist with Randy, to which he would always have something thoughtful to add. Randy’s mentorship helped me publish a few articles with him in journals like *PLoS ONE* [1] and *Clinical Immunology* [2] based on the Affymetrix microarray analyses combined with a multiplex protein array system for cytokines and chemokines, which for the first time highlighted the robust upregulation of various innate immune genes that are mainly involved in antiviral immune response, phagolysosome maturation, and MHC class-II expression in murine β-coronavirus infected chronically inflamed tissues. My wholehearted thanks to him on behalf of me and my student, for his immense help and contribution in this project which also led to the Ph.D. thesis of Dr. Kaushiki Biswas, her gratitude towards Dr. Cohrs is evident from her anecdote quoted below (Box 1).

Box 1 **Anecdote from Dr. Kaushiki Biswas, Assistant Professor at Department of Biological Sciences, Presidency University, Kolkata, India**

I had the privilege to meet Prof. Randall J Cohrs when I was a Ph.D. student, during the Indo-US symposium on Viral Infections of the Nervous System, 2014 at Gurgaon, India. I was working in the laboratory of Prof. Jayasri Das Sarma and was in my final year of Ph.D. As he was eager to interact with the young minds, an exclusive interaction session was arranged for us where we got the opportunity to have a direct meeting with him on a round table. Prof. Cohrs interacted with all the students and research scholars with curiosity and patience. He illuminated us with his profound knowledge of viruses and mentored us to a career in science. When I look back, I want to thank him for his thoughtful gesture, which also reflects a part of his persona. All throughout my PhD career his scientific publications on viruses acted as a reference and guiding path to my work. He had also provided his scientific insights into my PhD dissertation. I am proud to share a publication with him as a co-author [2].

In 2014, one of my BS-MS students, Mr. Jibin Sadasivan (Box 2), received the Du Pré grant, which was awarded to young MS researchers from the Multiple Sclerosis International Federation (MSIF), United Kingdom, to make short visits to established MS research centres outside of their own country. Randy was his host for the program. Jibin was indeed overwhelmed by Randy’s mentorship style, reflected in his testimony.

Box 2 **Anecdote from Mr. Jibin Sadasivan, Graduate Student at University of British Columbia, Vancouver, Canada**

I met Randy in 2014 when I was doing a short internship in his lab at the University of Colorado. Moving to a new country for the first time wasn’t easy, but Randy made sure that I had a smooth transition and that I did not feel left alone during my time there. He drove me to the lab every day and took me on short trips to Denver and Boulder on weekends. He made sure that I got souvenirs and pictures from the places we visited together. After my internship, we stayed in touch, and he continued to mentor me as I moved to grad school. Randy was a passionate mentor, a fantastic friend, and an inspiration to many of us, personally and professionally. I will be forever honoured and grateful to have had the opportunity to know him and work with him.

Prof. Gilden urged me to host the Indo-US bilateral symposium in India; this would facilitate cultural exchange in addition to science communication. With his support, we hosted a symposium in 2014 in New Delhi with Dr. Pankaj Seth (Indian Host), myself (Indian Co-Host) and Randy (US-Host), and Dr. Lynn Pullium (US Co-Host). The meeting received a great response. Dr. Randall Cohrs, Dr. Ravi Mahalingam, and others presented their work. Dr. Donald Gilden delivered a plenary lecture on temporal arteritis, which the audience received with overwhelming enthusiasm. My students got a chance to interact with Randy here for the first time, and they were overwhelmed with Randy’s easy-going nature and the vital perspective that he gave them on their research projects. After this meeting, our group of scientists took a golden triangle tour to the cities of Agra, Jaipur, and Delhi to experience the culture of these Indian cities (Figure 2).

Prof. Donald Gilden was also looking forward to the next Indo US bilateral symposium I hosted in 2016, but he could not attend the meeting due to his sad demise. Dr. Cohr continued to support my endeavours to conduct meetings. He was the Co-PI for the Bioanalytical tools and techniques workshop (BAW) 2018, held around the Indian festival of Colours, Holi (Figure 3 and Figure 4). This festival celebrates the eternal and divine love of Radha Krishna and signifies the triumph of good over evil. Randy enjoyed playing Holi with colours, and “work hard, play hard” was his slogan for the students during this meet. The students thoroughly enjoyed his company during the festival and the meeting (Figure 5). Randy also urged me to apply for ASMCUE 2017, where I received the American Society for Microbiology Undergraduate Leadership Award. In 2019, he invited me to the 19th Rocky Mountain Virology Association Meeting, held at a captivating and picturesque Mountain Campus of Colorado State University at the Pingree Park. He gave me an opportunity to deliver the invited opening presentation on the protective role of Ifit2 during neurotropic virus infection, followed by a keynote lecture by Dr. W. Ian Lipkin. Randy was always up-to date with the current advances in Virology and was an enthusiastic for hosting people with upcoming and important studies.

Randy was certainly a great leader. His leadership quality is evident from how he established and successfully conducted meetings for the Colorado Alpha Herpesvirus Latency Society (CALS) and led the European Society for Translational Medicine as the Vice-president and Rocky Mountain Virology Association (RMVA) as president.

In 2020, amidst the pandemic, we hosted the Indo US bilateral symposium in virtual mode on COVID biology, which hosted many experts from the field and led to interesting discussions on the outbreak and evolution of coronaviruses. He taught me that to be a good scientist, it is crucial to be a good mentor. The last meeting that I hosted with his guidance was the National Association of Biology Teachers (NABT) meeting in 2021, titled “Tips for Teaching Science in a Pandemic: Ways to Lessen the Stress on both Students and Educators”. This series was presented under the banner of NABT and supported by the Indian Institute of Science Education and Research (IISER Kolkata), the Indian Institute of Science (IISc Bangalore), the University of Colorado-School of Medicine and Northwestern Connecticut Community College, USA. Randy was very enthusiastic about having insights regarding the rich Indian tradition of learning and education that prevails since ancient times, The Gurukul System. The Gurukul System is a residential schooling system whose origin dates back to around 5000 BC in the Indian subcontinent. It was more prevalent during the Vedic age where students were taught various subjects and about how to live a cultured and disciplined life. Gurukul was actually the home of the teacher or Acharya and was the centre of learning where pupils resided until their education was completed. At the Gurukul, all were considered equal, and the guru (teacher) as well as shishya (student) resided in the same house, following a system of stages of life discussed in Hindu texts of the ancient and medieval eras. These four stages are: Brahmacharya (student), Gṛhastha (householder), Vanaprastha (forest walker/forest dweller), and Sannyasa (renunciate). The Asrama system is one important facet of the Dharma concept in Hinduism. Randy was overwhelmed with the practicality of this four stages of life.

In 2020, IUSSTF forum commenced a call-for-proposal for a collaborative effort involving scientists from India and US to explore the therapeutic targets to reduce SARS-CoV-2 infectivity. With the Principal Investigator from the US, Dr. Maria Nagel, I formed a group of scientists including Randy, who worked together to explore the efficacy of *Azadirachta indica* A. Juss (Neem) bark extract in restricting β-Coronaviral infection and replication. Randy played an active role in this project, and his experience in virology helped us throughout the project [3] (Figure 6). It is deeply saddening that we lost Randy. He was an inspiration and kept us all motivated.

The testimonies from all the students signify the friendly and warm personality of Dr. Cohrs (Box 3). His sad demise is a great loss for all of us. Randy was a genuine, kind, and compassionate person, and an inspiration to many. In his honour, at the BAW 2022 that I will be hosting, I am organizing a “Professor Randall J. Cohrs Memorial Lecture”. I shall miss his guidance and presence, and I hope that he continues to support us in spirit. All my current students and alumni miss you Randy! However, we know that you are always with us.

Box 3 **Anecdotes from Students and Alumni of my Lab who met Prof. Randall Cohrs**

*Dr. Lucky Sarkar, Post-Doctoral Fellow, Florida Research & Innovation Center, Cleveland Clinic, Florida, USA.*

During my Ph.D. days, in February 2018, while working under Prof. Jayasri Das Sarma at IISER Kolkata, I had the privilege of interacting with Prof. Randall J. Cohrs in an International INDO-US Biological and Analytical Workshop, Gangtok, India. Before that, I had heard a lot about his work, personality, knowledge, and sense of humour from my mentor Prof. Das Sarma. Undoubtedly, he had a great love not only for Science but also for different cultures and traditions. He used to resemble our Bengali polymath and Nobel Laureate Rabindranath Tagore. He was always enthusiastic about meeting young research minds and take part in cultural programs like dance or music. He also encouraged me to participate in cultural programs like NABT and other national and international platforms. I was fortunate enough to work in an IUSSTF-funded virtual networking grant on COVID-19 since the pandemic struck the world. Since April 2020, we have been working hard on our project “*Azadirachta indica* A. Juss (Neem) bark extract and its Nimbin isomers restrict β-Coronaviral infection and replication”, and drafting our manuscript together with other collaborators from India, USA, and also from Sweden. However, we suddenly lost such an eminent scientist and a humble soul one year before our paper was published in *Virology*, Elsevier, 2022. Dr. Cohrs has made one of the most tangible contributions to my research life. We miss you, Sir.

*Dr. Abhishek Bose, Post-Doctoral Fellow, Department of Genetic Medicine at Weill Cornell Medicine, New York, USA.
*

During the PhD period, I was fortunate enough to get several chances to meet Dr. Randall Cohrs and it was my absolute privilege and honour to know him from a close distance. We met in the Indo -US science and technology forum (IUSSTF) conference and the BAW-bioanalytical methods workshop organized by Prof. Das Sarma in 2018. In the meeting, I not only got a chance to discuss Science but also interacted with Prof. Cohrs like a friend. Not only was he a renowned virologist and neurologist, but also one who would make us feel at home with his warm smile and jovial nature. He took active part in the cultural program organized as a part of the conference and also met and dressed like the local people of Sikkim. Later, when I joined a post-doctoral position in IUSSTF networking project on COVID-19 with Prof. Das Sarma, we enjoyed Prof. Cohrs’s presence as a collaborator of the team, where he provided thoughtful scientific insights to steer the project at its inception. Also, he made an appearance in our spiritual forum “Kathanubhuti”, where he listened to the glories of Indian cultural heritage and slokes from Srimad Bhagavat Gita with deep interest and shared his spiritual knowledge and love for the Hindu religion. His inclusive and kind nature will keep him alive bright as sunshine, among all of us, who remain inspired by him. We miss you Prof. Cohrs (fondly known as Randy), as a teacher, friend and philosopher, as someone I will always look up to. My prayers for you and for your loving family.

## Figures and Tables

**Figure 1:**
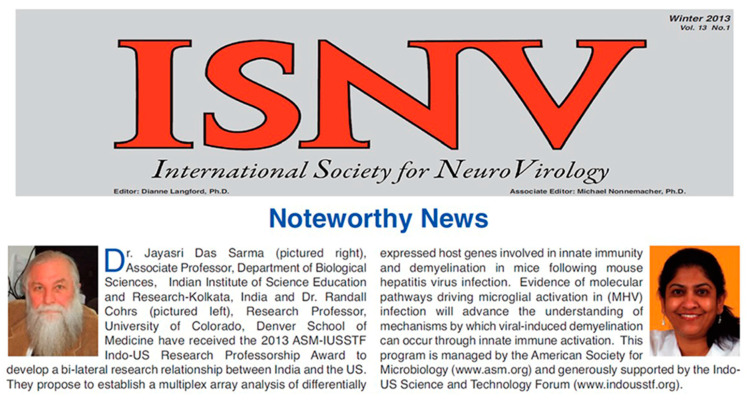
**ASM-IUSSTF Indo-US Research Professorship award.** The ASM-IUSSTF Indo-US research Professorship award for the year 2013 was awarded to Dr. Jayasri Das Sarma, with Dr. Cohrs as her mentor. The news was highlighted in the ISNV newsletter.

**Figure 2:**
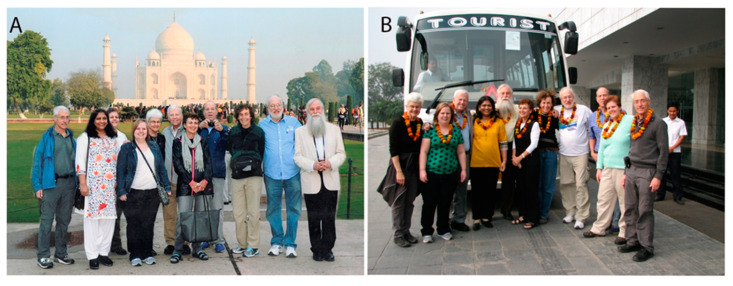
**Meeting in mind and travel in heart.** Virologists on a tour of the golden triangle after the Indo US bilateral symposium held in India in 2014. (**A**) Visit to the Taj Mahal. (**B**) Traditional Indian welcome of guests with garland. Besides Randy, the picture includes Dr. Stanley Perlman, Dr. Susan Wiess, Dr. Donald Gilden, Dr. Howard Lipton, Mr. Ed, Ms. Audry Gilden, Mrs. Pam Lipton, Dr. Kathryn Iacono, Dr. Jayasri Das Sarma, and Ms. Kimberly Dine.

**Figure 3:**
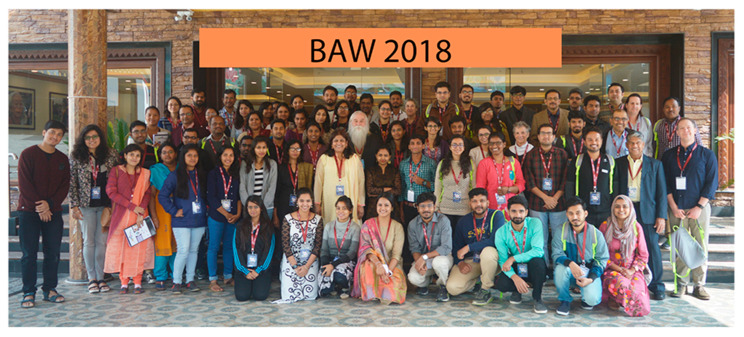
**The Bioanalytical tools and techniques workshop (BAW) 2018.** Under the mentorship of Dr. Cohrs, we hosted the workshop, which received a great response. The picture depicts the entire group that attended BAW 2018, with Randy and me in the centre, along with Dr. Ravi, Dr. Seth, Dr. Brent, Dr. Patricia, and Dr. Sue.

**Figure 4:**
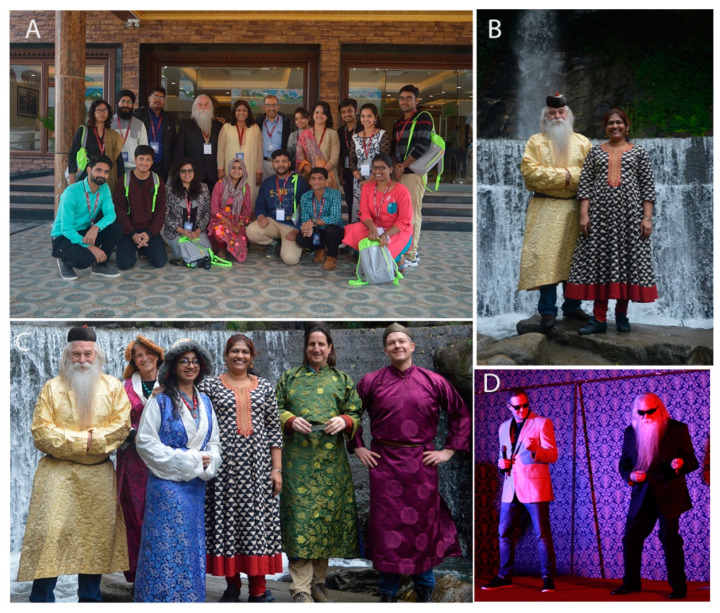
**Fun times at BAW 2018.** (**A**) COVID biology lab (IISER Kolkata) group with Dr. Cohrs and Dr. Ravi Mahalingam. (**B**,**C**) Fun outing to a nearby waterfall during the workshop. (**C**) Randy, Dr. Palmer and Dr. Seth dressed in traditional Sikkim attire. (**D**) Randy and Dr. Seth’s performance at the cultural program.

**Figure 5:**
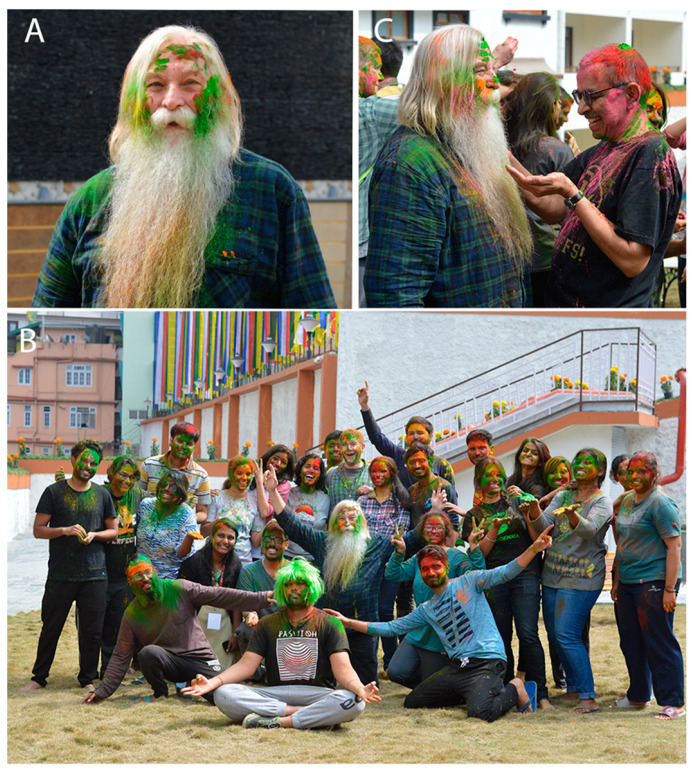
**Soaked in colour.** (**A**) Randy covered in Holi colours. (**B**) When a colleague becomes your dearest friend and brother; Dr. Ravi Mahalingam and Randy playing with Holi colours. (**C**) Randy enjoying Holi with the students during BAW 2018.

**Figure 6:**
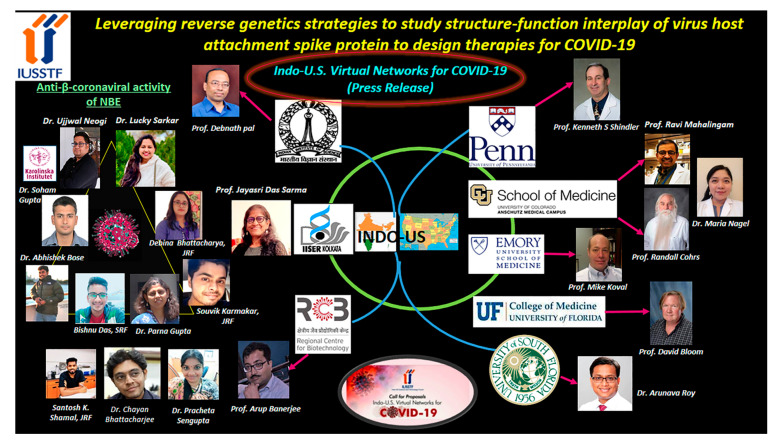
**The Indo-US virtual network project.** The picture depicts the entire team of scientists from India, US, and other parts of the world working together to understand the anti-β-coronaviral activity of NBE. Randy was a significant part of the group, and his absence will be felt forever.
